# Associations Between the Severity of Influenza Seasons and Mortality and Readmission Risks After Elective Surgical Aortic Valve Replacement and Coronary Artery Bypass Graft Surgery in Older Adults

**DOI:** 10.1001/jamanetworkopen.2020.31078

**Published:** 2020-12-23

**Authors:** Makoto Mori, Yun Wang, Shiwani Mahajan, Arnar Geirsson, Harlan M. Krumholz

**Affiliations:** 1 Center for Outcomes Research and Evaluation, Yale New Haven Hospital, New Haven, Connecticut; 2 Richard and Susan Smith Center for Outcomes Research in Cardiology, Division of Cardiology, Beth Israel Deaconess Medical Center, Harvard Medical School, Boston, Massachusetts; 3 Section of Cardiovascular Medicine, Department of Internal Medicine, Yale School of Medicine, New Haven, Connecticut; 4 Division of Cardiac Surgery, Yale School of Medicine, New Haven, Connecticut; 5 Department of Health Policy and Management, Yale School of Public Health, New Haven, Connecticut

## Abstract

This cross-sectional study uses Centers for Medicare and Medicaid Services claims data to investigate associations between influenza season severity and mortality and readmission risks after elective surgical aortic valve replacement (SAVR) and coronary artery bypass grafting (CABG) among older adults.

## Introduction

Patients recovering from a major operation are increasingly susceptible to respiratory complications, but whether undergoing operations during more severe influenza seasons is associated with a higher risk of respiratory complications remains unknown.^1,2,3^ Because elective procedures may be postponed to mitigate the potential risk, we evaluated associations between the severity of influenza seasons and the risks of mortality and hospital readmissions after elective coronary artery bypass grafting (CABG) and surgical aortic valve replacement (SAVR).

## Methods

We used Medicare fee-for-service inpatient claims data from the Centers for Medicare and Medicaid Services to identify patients 65 years or older who underwent elective isolated CABG or SAVR during influenza seasons between November 1 and March 31 of the years 2003 through 2017. Analyses were conducted in March 2 through May 23, 2020. According to the Centers for Disease Control and Prevention definitions, the duration and severity of influenza seasons were defined as mild, moderate, and severe based on rates of influenza-associated hospitalizations and deaths related to pneumonia or influenza.^4^ Outcomes were in-hospital and 30-day mortality and all-cause and pneumonia-related 30-day readmissions. Comparing outcomes between influenza seasons of different severity controlled for other potential seasonal variations in outcomes. Operations were identified based on the *International Classification of Diseases, Ninth Revision* and *International Statistical Classification of Diseases and Related Health Problems, Tenth Revision* codes (eTable in the Supplement). The comorbidities were defined according to the Hierarchical Condition Categories selected to capture important variations in cardiovascular disease outcomes.^5^ The Yale University Institutional Review Board approved the study and waived individual consent because of the minimal risk nature of the study.

We fitted a mixed model with a logit link function for mortality outcomes and a Cox regression model for readmission outcomes to evaluate the association with the seasonal influenza severity, adjusting for demographic characteristics and comorbid conditions (eAppendix in the Supplement). Readmission risks were modeled with competing risk of death.^6^ We used SAS software, version 9.4 (SAS Institute Inc). Statistical tests were 2-sided, and *P* < .05 was considered statistically significant. The study followed the guidelines for cohort studies in the Strengthening the Reporting of Observational Studies in Epidemiology (STROBE) Statement.

## Results

Of the influenza seasons studied, 6 were mild, 5 were moderate, and 4 were severe.^4^ Among 313 159 and 135 550 individuals who underwent CABG and SAVR procedures, respectively, the median age was 73 years (interquartile range, 69-78 years) in the CABG group (of whom 222 056 [70.9%] were men) and 76 years (interquartile range, 71-81 years) in the SAVR group (82 607 [60.9%] were men). Table 1 presents the patient characteristics according to the severity of influenza season.

**Table 1.** Patient Characteristics for CABG and SAVR Procedures by Severity of Influenza Seasons

Characteristic	Severity of influenza season by procedure group, No. (%)^a^					
	CABG			SAVR		
	Mild (n = 129 066)	Moderate (n = 105 464)	Severe (n = 78 629)	Mild (n = 54 944)	Moderate (n = 45 895)	Severe (n = 34 711)
Age, median (IQR), y	74 (69-78)	73 (69-78)	73 (69-78)	76 (71-81)	76 (71-81)	75 (70-80)
Male	91 118 (70.6)	74 718 (70.8)	56 220 (71.5)	33 232 (60.4)	27 967 (60.9)	21 408 (61.7)
Heart failure	13 168 (10.2	10 542 (10.0)	8080 (10.3)	8954 (16.3)	7303 (15.9)	5037 (14.5)
Myocardial infarction	8240 (6.4	6869 (6.5)	5541 (7.0)	1804 (3.3)	1597 (3.5)	1153 (3.3)
Unstable angina	5749 (4.5)	4692 (4.4)	3731 (4.7)	1373 (2.5)	1196 (2.6)	853 (2.5)
Atherosclerosis	72 038 (55.8)	57 605 (54.6)	42 500 (54.1)	30 866 (56.2)	25 065 (54.6)	18 225 (52.4)
Peripheral vascular diseases	9789 (7.6)	7519 (7.1)	5465 (7.0)	4368 (7.9)	3496 (7.6)	2526 (7.3)
Hypertension	82 051 (63.6)	66 452 (63.0)	47 900 (60.9)	31 345 (57.0)	26 218 (57.1)	18 912 (54.5)
Diabetes	42 542 (33.0)	35 103 (33.3)	26 964 (34.3)	13 985 (25.5)	11 659 (25.4)	9097 (26.2)
Stroke	1455 (1.1)	1263 (1.2)	949 (1.2)	561 (1.0)	450 (1.0)	383 (1.1)
Cerebrovascular disease	6220 (4.8)	5021 (4.8)	3699 (4.7)	2390 (4.3)	1969 (4.3)	1378 (4.0)
Respiratory failure	3275 (2.5)	2710 (2.6)	2247 (2.9)	1691 (3.1)	1510 (3.3)	1172 (3.4)
Asthma	2931 (2.3)	2563 (2.4)	1883 (2.4)	1593 (2.9)	1349 (2.9)	1071 (3.1)
COPD	23 204 (18.0)	18 721 (17.8)	13 098 (16.7)	8814 (16.0)	7174 (15.6)	5029 (14.5)
Pneumonia	8094 (6.3)	6410 (6.1)	4730 (6.0)	4041 (7.4)	3274 (7.1)	2297 (6.6)
Kidney failure	17 541 (13.6)	14 159 (13.4)	10 627 (13.5)	7813 (14.2)	6545 (14.3)	4935 (14.2)
Dementia	1761 (1.4)	1415 (1.3)	1061 (1.3)	857 (1.6)	696 (1.5)	510 (1.5)
Depression	4082 (3.2)	3468 (3.3)	2711 (3.4)	1880 (3.4)	1576 (3.4)	1293 (3.7)
Psychiatric diseases	1199 (0.9)	920 (0.9)	738 (0.9)	520 (0.9)	402 (0.9)	325 (0.9)
Parkinson or Huntington disease	699 (0.5)	612 (0.6)	443 (0.6)	321 (0.6)	273 (0.6)	209 (0.6)
Blood loss anemia	45 920 (35.6)	39 760 (37.7)	32 860 (41.8)	22 299 (40.6)	20 005 (43.6)	17 005 (49.0)
Trauma	3488 (2.7)	2540 (2.4)	1833 (2.3)	1704 (3.1)	1352 (2.9)	908 (2.6)
Protein-calorie malnutrition	3028 (2.3)	2394 (2.3)	1887 (2.4)	1669 (3.0)	1405 (3.1)	1049 (3.0)
Functional disability	919 (0.7)	830 (0.8)	666 (0.8)	320 (0.6)	308 (0.7)	232 (0.7)
Outcomes, 30 d						
Readmission						
Pneumonia related	577 (0.4)	458 (0.4)	328 (0.4)	311 (0.6)	253 (0.6)	192 (0.6)
All cause	20 300 (15.7)	16 188 (15.3)	11 046 (14.0)	9727 (17.7)	7828 (17.1)	5336 (15.4)
Mortality	3976 (3.1)	3347 (3.2)	2452 (3.1)	2286 (4.2)	1868 (4.1)	1297 (3.7)

Abbreviations: CABG, coronary artery bypass grafting; COPD, chronic obstructive pulmonary disease; IQR, interquartile range; SAVR, surgical aortic valve replacement.

^a^ The severity of influenza seasons was categorized on the basis of influenza-associated hospitalization and mortality related to pneumonia or influenza.^4^

Compared with CABG procedures during mild influenza seasons, those performed during moderate (odds ratio [OR], 1.06; 95% CI, 1.01-1.11) and severe (OR, 1.06; 95% CI, 1.01-1.12) influenza seasons had higher associated risks of 30-day mortality. In SAVR procedures, the 30-day mortality risk was not significantly higher in moderate (OR, 1.03; 95% CI, 0.96-1.10) or severe (OR, 1.01; 95% CI, 0.94-1.06) influenza seasons compared with mild influenza seasons. All-cause readmission risk was not statistically significantly higher in CABG or SAVR procedures performed during influenza seasons of moderate severity (OR, 0.99; 95% CI, 0.97-1.01 for CABG; OR, 0.99; 95% CI, 0.96-1.02 for SAVR) or severe influenza seasons (OR, 0.92; 95% CI, 0.90-0.95 for CABG, OR, 0.93; 95% CI, 0.90-0.96 for SAVR) compared with influenza seasons of mild severity (Table 2).

**Table 2.** Risk of 30-Day Mortality and Readmissions After CABG and SAVR Procedures by Severity of Influenza Seasons^a^

Outcome	Influenza severity	HR or OR (95% CI)^b^	*P* value
**Coronary artery bypass graft**			
In-hospital mortality	Severe	1.06 (1.00-1.12)	.048
	Moderate	1.06 (1.01-1.11)	
	Low	1 [Reference]	
30-d Mortality	Severe	1.06 (1.01-1.12)	.03
	Moderate	1.06 (1.01-1.11)	
	Low	1 [Reference]	
30-d All-cause readmission	Severe	0.92 (0.90-0.95)	<.001
	Moderate	0.99 (0.97-1.01)	
	Low	1 [Reference]	
30-d Pneumonia-related readmission	Severe	0.95 (0.84-1.07)	.66
	Moderate	0.97 (0.87-1.07)	
	Low	1 [Reference]	
**Surgical aortic valve replacement**			
In-hospital mortality	Severe	1.02 (0.95-1.10)	.89
	Moderate	1.01 (0.94-1.07)	
	Low	1 [Reference]	
30-d Mortality	Severe	1.01 (0.94-1.08)	.66
	Moderate	1.03 (0.96-1.10)	
	Low	1 [Reference]	
30-d All-cause readmission	Severe	0.93 (0.90-0.96)	<.001
	Moderate	0.99 (0.96-1.02)	
	Low	1 [Reference]	
30-d Pneumonia-related readmission	Severe	0.91 (0.75-1.09)	.39
	Moderate	1.03 (0.89-1.20)	
	Low	1 [Reference]	

Abbreviations: CABG, coronary artery bypass grafting; HR, hazard ratio; OR, odds ratio; SAVR, surgical aortic valve replacement.

^a^ The severity of influenza seasons was categorized on the basis of influenza-associated hospitalization and mortality related to pneumonia or influenza.^4^

^b^ The hazard ratio is used for 30-day all-cause and pneumonia-related readmission risks; the odds ratio is for in-hospital and 30-day mortality. The hazard and odds ratios are both risk adjusted for demographic characteristics and comorbid conditions.

## Discussion

Our findings showed that, among older adults who underwent elective coronary and aortic valve operations during influenza seasons, there were no consistent associations between the severity of the influenza season and the risk of mortality or readmission. For CABG procedures, increasing seasonal severity may be associated with a slightly higher risk of 30-day mortality. Limitations of this study include claims-based data that limited granularity used in characterizing the severity of pneumonia and operative risks. Geographic variation in the severity of influenza seasons was not accounted for. Our large sample suggests that it is safe to perform elective major operations regardless of the severity of influenza seasons.

## Notes

Supplement. **eAppendix.** Model Specifications **eReferences.** **eTable.** ICD-9 and -10 Codes for Surgical Aortic Valve Replacement, Transcatheter Aortic Valve Replacement, Coronary Artery Bypass Graft Surgery, and Percutaneous Coronary Intervention
