# Supplementary material for: Associations Between the Severity of Influenza Seasons and Mortality and Readmission Risks After Elective Surgical Aortic Valve Replacement and Coronary Artery Bypass Graft Surgery in Older Adults
Source: JAMA Netw Open. 2020 Dec 23;3(12):e2031078. doi: 10.1001/jamanetworkopen.2020.31078 (PMC7758803; doi:10.1001/jamanetworkopen.2020.31078)
Supplement: Supplement. — eAppendix. Model Specifications eReferences. eTable. ICD-9 and -10 Codes for Surgical Aortic Valve Replacement, Transcatheter Aortic Valve Replacement, Coronary Artery Bypass Graft Surgery, and Percutaneous Coronary Intervention [file jamanetwopen-e2031078-s001.pdf]

Supplemental Online Content

Mori M, Wang Y, Mahajan S, Geirsson A, Krumholz HM. Associations between the severity of influenza seasons and mortality and readmission risks after elective surgical aortic valve replacement and coronary artery bypass graft surgery in older adults. *JAMA Netw Open*. 2020;3(12):e2031078. doi:10.1001/jamanetworkopen.2020.31078

eAppendix. Model Specifications

eReferences

eTable. ICD-9 and -10 Codes for Surgical Aortic Valve Replacement, Transcatheter Aortic Valve Replacement, Coronary Artery Bypass Graft Surgery, and Percutaneous Coronary Intervention

This supplemental material has been provided by the authors to give readers additional information about their work.

© 2020 Mori M et al. *JAMA Network Open.*

eAppendix. Model Specifications

Both mixed model with logit link function Cox proportional hazard model were fitted on the same set of demographic and comorbidity variables described in Table 1. We also included a time variable, ranging from 0 (year 2003) to 14 (year 2017) in models to account for secular trends in outcomes during the study period, after demonstrating that the incidences of all 4 outcomes conformed to linear trends over the study period. In-hospital and 30-day mortality risks were modeled using a mixed model while readmission outcomes were modeled with Cox model accounting for competing risk of death. Therefore, there were 4 models per case type, fitted for each of the 4 outcomes. We estimated these models with hospital-specific random intercepts to account for within-hospital and between-hospital. The covariates were introduced simultaneously without further variable selection, as the list of variables were generated from hierarchical conditional categories specifically selected for predicting outcomes of cardiovascular diseases in prior works from Centers for Medicare and Medicaid measure development.

Proportional hazard model assumption was checked using Schoenfeld residuals test,[1] which indicated that the categorical variable represented severity of influenza seasons met the proportional hazards assumption for the readmission outcomes for both CABG and SAVR conditions. Some variables (unstable angina, protein-calorie malnutrition, age, sex, HTN, and Parkinson/Huntington disease) that did not meet the assumption, which may be related to the large sample size. The coefficients for these variables were interpreted to represent an average effect over the 30-day period.[2]

© 2020 Mori M et al. *JAMA Network Open.*

eReferences

1. Schoenfeld D. Partial residuals for the proportional hazards regression model. *Biometrika*. 1982;69(1):239-241.

2. Stensrud MJ, Hernán MA. Why Test for Proportional Hazards? *JAMA*. 2020;323(14):1401-1402.

eTable. ICD-9 and -10 codes for surgical aortic valve replacement, transcatheter aortic valve replacement, coronary artery bypass graft surgery, and percutaneous coronary intervention

SAVR			CABG	
**ICD9**	**ICD10**		**ICD9**	**ICD10**
3521	02RF08Z		36.10	0210083
3522	02RF0JZ		36.11	0210088
	02RF07Z		36.12	0210089
	02RF0KZ		36.13	0210093
	X2RF032		36.14	0210098
			36.15	0210099
TAVR			36.16	0210483
**ICD9**	**ICD10**		36.17	0210488
3505	02RF37H		36.19	0210489
3506	02RF38H			0210493
	02RF3JH			0210498
	02RF3KH			0210499
	02RF37Z			0211083
	02RF38Z			0211088
	02RF3JZ			0211089
	02RF3KZ			0211093
	X2RF332			0211098
				0211099
PCI				0211483
**ICD9**	**ICD10**			0211488
3601	270346			0211489
3602	027034Z			0211493
3605	027034Z			0211498
3606	02703D6			0211499
3607	02703DZ			0212083
0066	02703Z6			0212088
	02703ZZ			0212089
	270446			0212093
	027044Z			0212098
	02704D6			212099
	02704DZ			0212483
	02724D6			0212488
	02724DZ			0212489
	02724Z6			0212493

© 2020 Mori M et al. *JAMA Network Open.*

	02724ZZ			0212498
	273346			0212499
	027334Z			0213083
	02734D6			0213088
	02734DZ			0213089
	02734Z6			0213093
	02734ZZ			0213098
				0213099
				0213483
				0213488
				0213489
				0213493
				0213498
				0213499
				021008C
				021008F
				021008W
				021009C
				021009F
				021009W
				02100A3
				02100A3
				02100A8
				02100A9
				02100AC
				02100AF
				02100AW
				02100J3
				02100J8
				02100J9
				02100JC
				02100JF
				02100JW
				02100K3
				02100K8
				02100K9
				02100KC
				02100KF
				02100KW

© 2020 Mori M et al. *JAMA Network Open.*

				02100Z3
				02100Z8
				02100Z9
				02100ZC
				02100ZF
				021048C
				021048F
				021048W
				021049C
				021049F
				021049W
				02104A3
				02104A8
				02104A9
				02104AC
				02104AF
				02104AW
				02104J3
				02104J8
				02104J9
				02104JC
				02104JF
				02104JW
				02104K3
				02104K8
				02104K9
				02104KC
				02104KF
				02104KW
				02104Z3
				02104Z8
				02104Z9
				02104ZC
				02104ZF
				021108C
				021108F
				021108W
				021109C
				021109F

© 2020 Mori M et al. *JAMA Network Open.*

				021109W
				02110A3
				02110A8
				02110A9
				02110AC
				02110AF
				02110AW
				02110J3
				02110J8
				02110J9
				02110JC
				02110JF
				02110JW
				02110K3
				02110K8
				02110K9
				02110KC
				02110KF
				02110KW
				02110Z3
				02110Z8
				02110Z9
				02110ZC
				02110ZF
				021148C
				021148F
				021148W
				021149C
				021149F
				021149W
				02114A3
				02114A8
				02114A9
				02114AC
				02114AF
				02114AW
				02114J3
				02114J8
				02114J9

© 2020 Mori M et al. *JAMA Network Open.*

				02114JC
				02114JF
				02114JW
				02114K3
				02114K8
				02114K9
				02114KC
				02114KF
				02114KW
				02114Z3
				02114Z8
				02114Z9
				02114ZC
				02114ZF
				021208C
				021208F
				021208W
				021209C
				021209F
				021209W
				02120A3
				02120A8
				02120A9
				02120AC
				02120AF
				02120AW
				02120J3
				02120J8
				02120J9
				02120JC
				02120JF
				02120JW
				02120K3
				02120K8
				02120K9
				02120KC
				02120KF
				02120KW
				02120Z3

© 2020 Mori M et al. *JAMA Network Open.*

				02120Z8
				02120Z9
				02120ZC
				02120ZF
				021248C
				021248F
				021248W
				021249C
				021249F
				021249W
				02124A3
				02124A8
				02124A9
				02124AC
				02124AF
				02124AW
				02124J3
				02124J8
				02124J9
				02124JC
				02124JF
				02124JW
				02124K3
				02124K8
				02124K9
				02124KC
				02124KF
				02124KW
				02124Z3
				02124Z8
				02124Z9
				02124ZC
				02124ZF
				021308C
				021308F
				021308W
				021309C
				021309F
				021309W

© 2020 Mori M et al. *JAMA Network Open.*

				02130A3
				02130A8
				02130A9
				02130AC
				02130AF
				02130AW
				02130J3
				02130J8
				02130J9
				02130JC
				02130JF
				02130JW
				02130K3
				02130K8
				02130K9
				02130KC
				02130KF
				02130KW
				02130Z3
				02130Z8
				02130Z9
				02130ZC
				02130ZF
				021348C
				021348F
				021348W
				021349C
				021349F
				021349W
				02134A3
				02134A8
				02134A9
				02134AC
				02134AF
				02134AW
				02134J3
				02134J8
				02134J9
				02134JC

© 2020 Mori M et al. *JAMA Network Open.*

				02134JF
				02134JW
				02134K3
				02134K8
				02134K9
				02134KC
				02134KF
				02134KW
				02134Z3
				02134Z8
				02134Z9
				02134ZC
				02134ZF

© 2020 Mori M et al. *JAMA Network Open.*
